# Investigation of clinically acceptable agreement between two methods of automatic measurement of limb occlusion pressure: a randomised trial

**DOI:** 10.1186/s42490-021-00053-9

**Published:** 2021-05-08

**Authors:** Luke Hughes, James McEwen

**Affiliations:** 1 Centre for Applied Performance Sciences, Faculty of Sport, Allied Health and Performance Sciences, St Mary’s University, London, UK; 2 Department of Orthopaedics, Faculty of Medicine, University of British Columbia, Vancouver, Canada

**Keywords:** Tourniquet, Limb occlusion pressure, Personalised, Tourniquet safety

## Abstract

**Background:**

Development of automatic, pneumatic tourniquet technology and use of personalised tourniquet pressures has improved the safety and accuracy of surgical tourniquet systems. Personalisation of tourniquet pressure requires accurate measurement of limb occlusion pressure (LOP), which can be measured automatically through two different methods. The ‘embedded LOP’ method measures LOP using a dual-purpose tourniquet cuff acting as both patient sensor and pneumatic effector. The ‘distal LOP’ method measures LOP using a distal sensor applied to the patient’s finger or toe of the operating limb, using photoplethysmography to detect volumetric changes in peripheral blood circulation. The distal LOP method has been used clinically for many years; the embedded LOP method was developed recently with several advantages over the distal LOP method. While both methods have clinically acceptable accuracy in comparison to LOP measured using the manual Doppler ultrasound method, these two automatic methods have not been directly compared. The purpose of this study is to investigate if the embedded and distal methods of LOP measurement have clinically acceptable agreement. The differences in pairs of LOP measurement in the upper and lower limbs of 81 healthy individuals were compared using modified Bland and Altman analysis. In surgery, it is common for cuff pressure to deviate from the pressure setpoint due to limb manipulation. Surgical tourniquet systems utilise a ± 15 mmHg pressure alarm window, whereby if the cuff pressure deviates from the pressure setpoint by > 15 mmHg, an audiovisual alarm is triggered. Therefore, if the difference (bias) ± SE, 95% CI of the bias and SD of differences ± SE in LOP measurement between the embedded and distal methods were all within ±15 mmHg, this would demonstrate that the two methods have clinically acceptable agreement.

**Results:**

LOP measurement using the embedded LOP method was − 0.81 ± 0.75 mmHg (bias ± standard error) lower than the distal LOP method. The 95% confidence interval of the bias was − 2.29 to 0.66 mmHg. The standard deviation of the differences ± standard error was 10.35 ± 0.49 mmHg. These results show that the embedded and distal methods of LOP measurement demonstrate clinically acceptable agreement.

**Conclusions:**

The findings of this study demonstrate clinically acceptable agreement between the embedded and distal methods of LOP measurement. The findings support the use of the embedded LOP method of automatic LOP measurement using dual-purpose tourniquet cuffs to enable accurate, effective and simple prescription of personalised tourniquet cuff pressures in a clinical setting.

## Introduction

Tourniquets are used to control venous and arterial blood flow in a limb to prevent life-threatening blood loss, create a bloodless surgical field, restrict intravenous local anaesthesia and, more recently, perform blood flow restriction therapy [9, 13, 21]. Clinical studies have shown that early generation tourniquets can result in temporary and permanent injury to underlying nerves and other tissues [13, 15] as a consequence of hazardously high tourniquet cuff pressures and applied pressure gradients [6, 14, 23]. Important advances in automatic, pneumatic tourniquet technology has improved the safety, accuracy and reliability of surgical tourniquet systems with the implementation of lower, controlled and personalised pressures with reduced applied pressure gradients [12].

A critical factor for safe and effective tourniquet cuff application is the use of personalised pressures based on a patient’s limb occlusion pressure (LOP) [13, 17]. LOP is defined as the minimum pressure required, at a specific time by a specific tourniquet cuff applied to a specific patient’s limb at a specific location, to stop the flow of arterial blood distal to the cuff [21]. Use of non-personalised, standard tourniquet pressures fails to account for several patient-specific variables, which often translates into the use of unnecessarily high pressures and high applied pressure gradients, increasing the risk of tissue injury [12, 22]. Measuring LOP significantly reduces the required pressure and applied pressure gradient compared to standard pressures [26], thereby reducing the likelihood of tourniquet-related injury [4, 12, 21, 26]. However, the clinical adoption of personalised tourniquet cuff pressures based on LOP has been limited by practical difficulties of LOP determination. The manual Doppler ultrasound method [7] can be used to measure LOP, and involves positioning a Doppler probe on a distal artery to monitor arterial blood flow distal to the applied tourniquet cuff by a trained and experienced operator. However, this method requires additional equipment, is time-consuming, and can be error-prone if performed by inadequately trained individuals [17, 24] and is therefore seldom used in clinical practice.

Consequential to advances in automatic, pneumatic tourniquet technology, a patient’s LOP can be measured automatically through two different methods. The first method, the ‘embedded LOP method’ [16], measures LOP by employing a dual-purpose tourniquet cuff to monitor arterial pulsations in an underlying limb by sensing pneumatic pressure pulsations in the cuff associated with volume changes in the limb as the cuff pressure is gradually increased [8, 12, 16]. A dual-purpose cuff is a specialised tourniquet cuff that acts as both patient sensor and pneumatic effector, as described in US Patent 8,425,551. The second method, the ‘distal LOP method’ [18, 19], measures LOP using a distal sensor applied to the patient’s finger or toe of the operative limb to monitor arterial pulsations as cuff pressure is gradually increased [18, 19]. The distal sensor uses photoplethysmography to detect volumetric changes in blood in peripheral circulation. LOP measured using the embedded method is referred to as ‘embedded LOP’ and LOP measured using the distal method is referred to as ‘distal LOP’. Both of these methods measure the same LOP and have each been shown to have clinically acceptable accuracy compared to the manual Doppler ultrasound method [12, 18, 19]. McEwen et al. [18] compared the accuracy of the distal LOP method to the manual Doppler ultrasound method, reporting a mean difference of 1.7 ± 8.9 mmHg between the two methods. Masri et al. [12] compared the accuracy of the embedded LOP method to the manual Doppler ultrasound method, reporting a mean difference of 1 ± 12 mmHg. Both of these studies concluded that the distal and embedded methods demonstrate clinically acceptable accuracy compared to the Doppler ultrasound method [12, 18].

The embedded LOP method offers several advantages over the distal LOP method. Primarily, the former circumvents the need for a separate, complex and costly distal sensor, which can affect the sterile field in a surgical setting. Moreover, placing and removing the distal sensor takes time which can affect the perioperative workflow. Finally, the success of LOP measurement using a distal sensor is dependent on variables affecting the measurement of low peripheral blood flow [16]. While both distal LOP and embedded LOP have each been compared to the manual Doppler ultrasound method [12, 18, 19], they have not been directly compared. If LOPs measured by embedded LOP and distal LOP demonstrate clinically acceptable agreement, this will support broader clinical usage of embedded LOP within surgical and therapy settings. Therefore, the purpose of this study is to investigate whether the embedded and distal methods of LOP measurement have clinically acceptable agreement.

## Methodology

### Participants

This study was conducted at the Medical Device Development Centre in Vancouver, Canada, between November–December 2020, using a tourniquet instrument capable of measuring LOP through both embedded LOP and distal LOP. For this study, 81 participants were recruited (Table 1). All participants were non-injured, non-smokers between the age of 18–75. Individuals were excluded if they presented with contraindications to tourniquet cuff use, vascular disease or circulation problems in the extremities, history of deep vein thrombosis, or inability to provide informed consent. All participants provided written informed consent in compliance with the Declaration of Helsinki [25]. This study was approved by St Mary’s University Research Ethics Committee (SMEC_2018-19_049).

**Table 1:** Participant anthropometric characteristics (Mean ± SD)

	n	Age (y)	Height (cm)	Body mass (kg)	Systolic blood pressure (mmHg)
Females	44	37 ± 16	162.89 ± 8.00	58.7 ± 10.9	113 ± 13
Males	37	42 ± 17	177.42 ± 6.16	79.8 ± 11.8	120 ± 8
Total	81	40 ± 16	169.53 ± 10.23	68.3 ± 15.4	116 ± 12

### Experimental design

#### Equipment

LOP measurement through embedded LOP and distal LOP involves the use of dual-purpose tourniquet cuffs and a tourniquet instrument containing pressure sensors and LOP measurement software algorithm. The dual-purpose tourniquet cuffs have a stiffened design that incorporates a continuous passageway that completely surrounds the underlying limb after application. For embedded LOP, measurement of LOP is completed using just the tourniquet cuff and the connected tourniquet instrument. For the distal method, a distal sensor is applied to the individual’s digit distal to the tourniquet cuff.

#### Experimental procedure

**Fig. 1:**
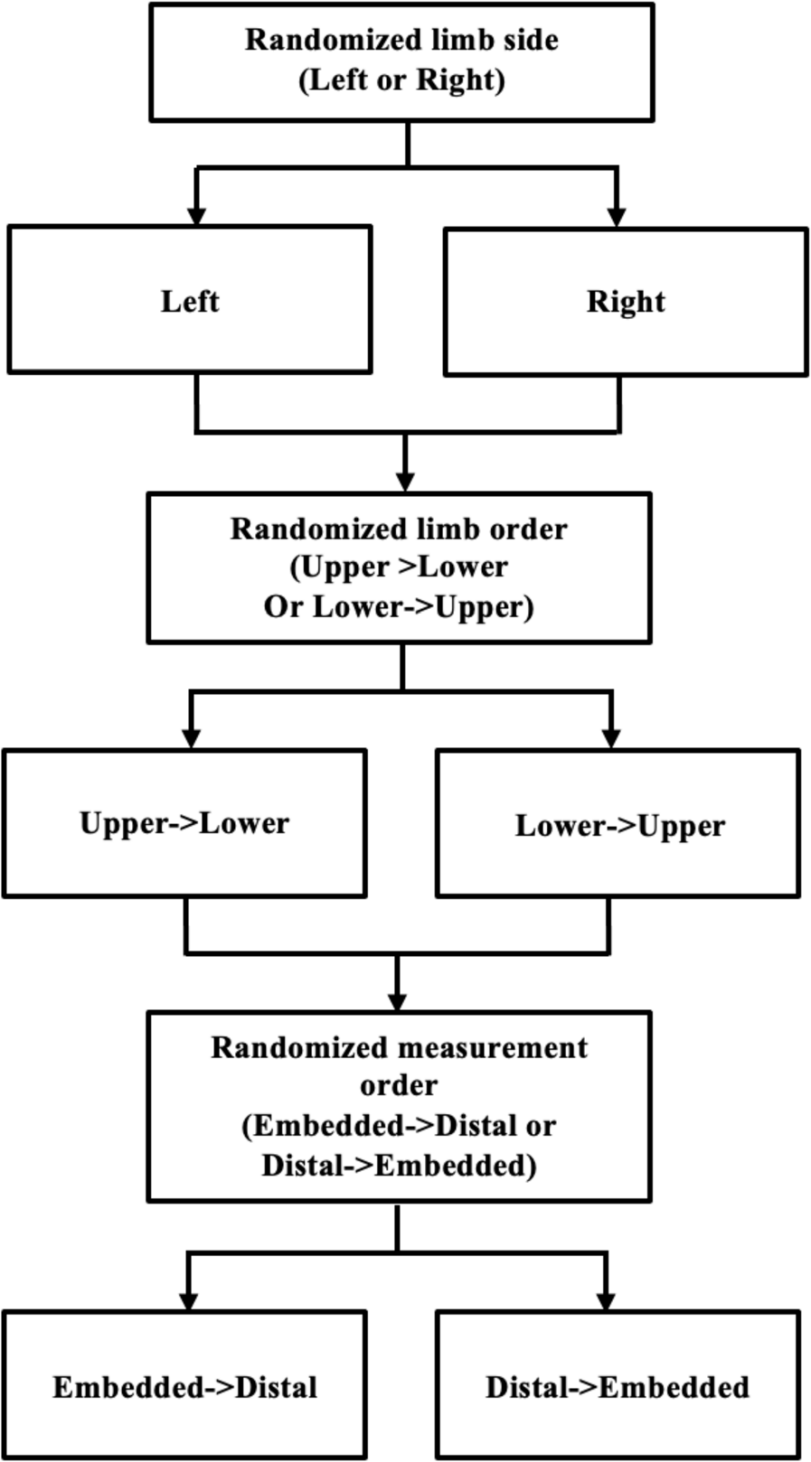
Randomisation procedure for limb and measurement order

A tourniquet cuff was applied to one upper and one lower limb on each participant. Both tourniquet cuffs were applied to the same side of the body (i.e. left or right), which was randomised prior to beginning data collection using a computerised random number generator (Fig. 1). Participants lay on a portable clinic bed and appropriately sized dual-purpose tourniquet cuffs were selected based on the manufacturer’s recommendations. The underlying matching limb protection sleeve was applied to the limb, followed by tourniquet cuff application. A blood pressure cuff connected to the CARESCAPE V100 Vital Signs Monitor (GE Healthcare, Buckinghamshire, UK) was applied to the contralateral upper limb. Blood pressure was measured at the beginning and end of the experimental sequence. Participants lay quietly still for 3 min to allow blood pressure to stabilise prior to the first measurement. If the individual felt cold, warm air was provided using a portable air warmer.

**Fig. 2:**
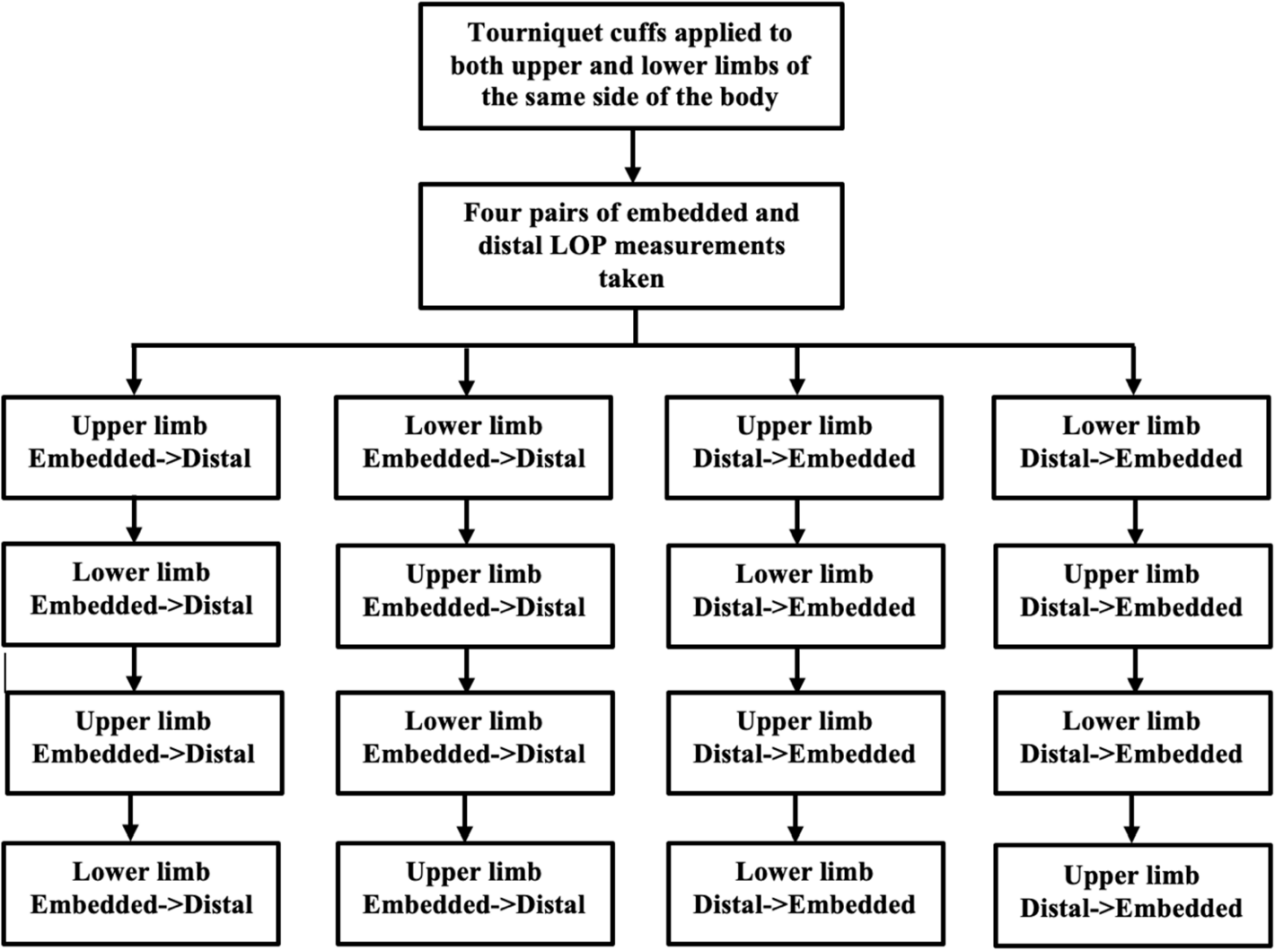
Overview of possible protocols following randomisation

On each tourniquet limb, two pairs of embedded and distal LOP measurements were taken. The order was randomised and used for all four measurement pairs (i.e. embedded-distal-embedded-distal or distal-embedded-distal-embedded) (Fig. 2). The first limb on which the initial pair of LOP measurements were performed was randomised, and subsequent pairs of measurement were done on alternating limbs (i.e. upper-lower-upper-lower or lower-upper-lower-upper) (Fig. 2). For distal LOP measurements, a distal sensor was applied to the individual’s index finger or second toe, which uses photoplethysmography to detect volumetric changes in blood in peripheral circulation. The distal sensor includes a LED acting as a quality indicator, providing feedback to the user on the quality of the physiologic signal from the distal sensor and a guide as to whether the LOP measurement will succeed or not based on the sensor application. This displays the signal qualities: 1) green (signal is good; measurement will complete); 2) yellow (signal is adequate; measurement will likely complete, but user should try to readjust distal sensor to obtain a green signal quality); and 3) red (signal is poor; measurement will not initiate, and user should adjust to try to obtain a green or yellow signal quality). The experimenter employed a maximum of 4 sensor adjustments to obtain a green signal, by reapplying the sensor on the same digit or on a different digit. If green or yellow signal quality was obtained, distal LOP measurement was initiated; if signal quality was red after 4 sensor adjustments, distal LOP measurement was not performed for that measurement pair. Signal quality was recorded manually. The tourniquet instrument increased the pressure in predetermined increments and LOP was defined as the pressure at which a distal pulse was no longer detected by the distal sensor.

For embedded LOP measurements, the tourniquet instrument increased the cuff pressure in predetermined increments, analysed the pneumatic pressure pulsations induced in the cuff bladder by the arterial pressure pulsations at each cuff pressure increment, and used these characteristics to determine LOP. In both measurements, the tourniquet cuff was immediately deflated after LOP was measured. LOP was displayed on the tourniquet instrument and recorded manually. The tourniquet instrument automatically discontinued a LOP measurement if the detected signal was too low or exceeded a certain limit during the measurement, and the experimenter discontinued the measurement if participants moved or talked during the measurement. A second measurement was attempted for any discontinued measurements; if this was successful, the second result was used, however, if unsuccessful, this measurement pair was excluded.

### Statistical analysis

The Bland and Altman method [11] was used to examine the agreement between the two methods of LOP measurement. LOP difference was defined as embedded LOP minus distal LOP. Due to repeated measurements on each individual, a modified approach was employed using a random effects model [20] to avoid underestimation of the variation of differences [1, 2]. The random effects model was used to estimate within-subject standard deviation (SD) while accounting for other observed and unobserved variations [10]. The sequence of measurement was used as the random effect, with inclusion of two potential explanatory variables as covariates: 1) mean measurement for each individual over time; and 2) the mean measurement between the two methods for each measurement occasion. To check the assumption that the differences were independent from the mean of the repeated measures and normally distributed, within-subject standard deviation (SD) was plotted against the mean of each individual by each method [1, 2, 20]. Within-subject SD was then used to create an appropriate Bland-Altman plot [2]. Limits of agreement (LOA) were established to assess the relative bias (mean difference) and random error (1.96 SD of the difference) between the embedded and distal methods. 95% confidence intervals (CI) were calculated to examine the estimation uncertainty for bias and LOAs [3, 5, 27]. The SD of differences ± standard error (SE), within- and between-subject variation ± SE were calculated [5]. Pressure variations within ±15 mmHg are common for pressure regulation during a surgical case. Surgical tourniquet systems utilise a ± 15 mmHg pressure alarm window [15]. If cuff pressure deviates from the reference pressure by > 15 mmHg, the surgical tourniquet system responds with audio-visual alarms. Therefore, a clinically acceptable difference of ±15 mmHg in LOP measurement was defined a priori. If the bias ± SE, SD of differences ± SE and 95% CI of the bias in LOP measurement between the embedded and distal methods were all within ±15 mmHg, this would demonstrate that the two methods have clinically acceptable agreement [12].

## Results

**Fig. 3:**
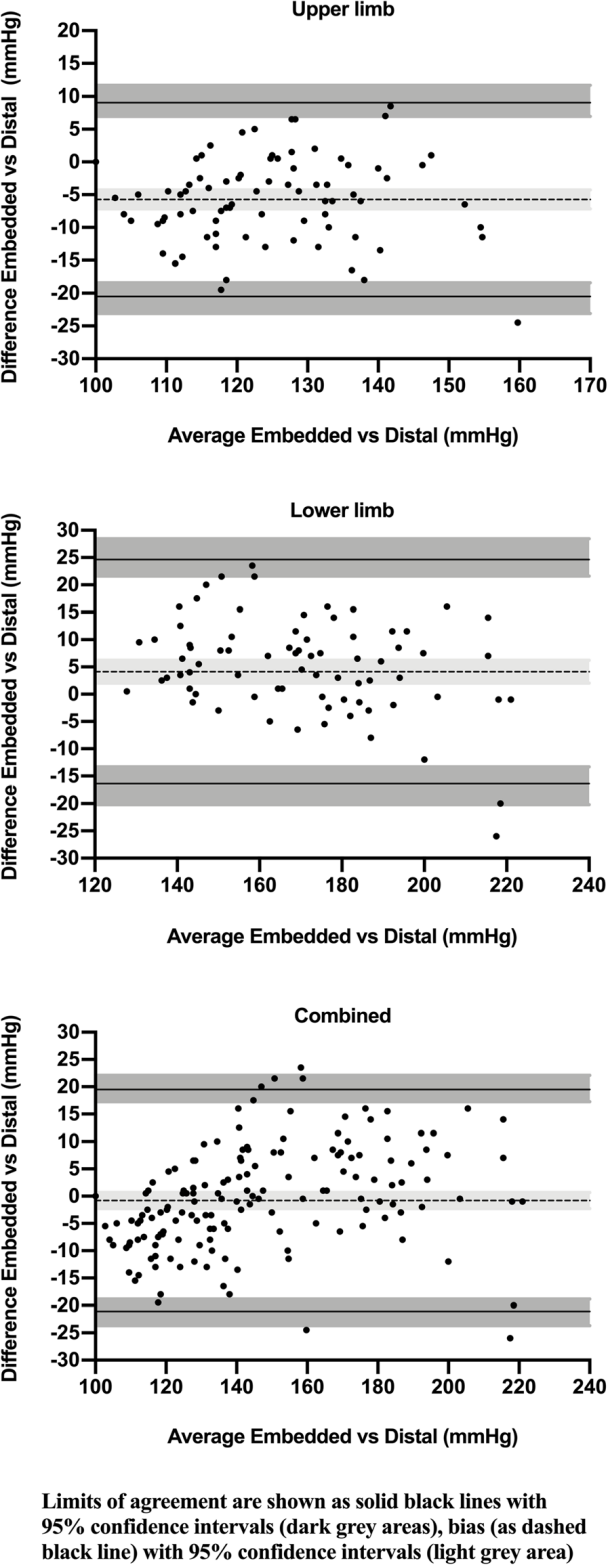
Bland-Altman plot of LOP differences

Reasons for exclusion of any measurements are detailed in Table 2. A histogram of the differences reflected normal distribution, and the scatter of the differences did not depend on the means, indicating homogeneity of variances across the measurement range [5]. Data are presented as mean ± SE with 95% CIs. LOAs with 95% CIs and SD of difference ± SE are detailed in Table 3. Bland and Altman plots of LOP differences between the embedded and distal methods are shown in Fig. 3. For all measurement in the upper and lower limbs combined, LOP measurement using the embedded method was − 0.81 ± 0.75 mmHg (95% CIs: − 2.29 to 0.66) lower compared to distal method. In the upper limb, LOP measurement using the embedded method was − 5.74 ± 0.73 mmHg lower (95% CIs: − 7.18 to − 4.29) compared to the distal method. In the lower limb, LOP measurement using the embedded method was 4.11 ± 1.05 mmHg (95% CIs: 3.19 to 7.02) higher compared to distal LOP. As the bias ± SE, SD of differences ± SE and 95% CI of the bias were within ±15 mmHg (Table 3), this demonstrates that the two methods of LOP measurement have clinically acceptable agreement.

**Table 2:** Discontinued measurements and data excluded from analysis

	Upper limb	Lower limb
Manually discontinued measurements		
Talking during measurement	–	1
Participant discomfort	1	–
Improper orientation of limb	2	–
Automatically discontinued measurements		
Unable to inflate to initial pressure	1	–
Low signal quality	–	2
Sensor off digit	3	4
Excluded measurement pairs		
Red signal quality	2	8

**Table 3:** Difference in LOP measurement between embedded and distal methods

	Upper	Lower	Combined
***N***	80	80	160
**Bias ± SE**	−5.74 ± 0.73	4.11 ± 1.05	−0.81 ± 0.75
**95% CI of bias**	−7.18 to −4.29	2.02 to 6.21	−2.29 to 0.66
**SD of difference ± SE**	7.54 ± 0.47	10.47 ± 0.69	10.35 ± 0.49
**LOA**	−20.51 to 9.04	−16.40 to 24.64	− 21.10 ± 19.48
**95% CI lower LOA**	−23.11 to −18.42	−20.21 to −13.33	−23.70 to − 18.86
**95% CI upper LOA**	6.94 ± 11.64	21.58 to 28.43	17.24 to 22.08

*LOP* limb occlusion pressure, *SD* standard deviation, *CI* confidence interval, *SE* standard error, *LOA* limits of agreement, *WSV* within-subject variation, *BSV* between-subject variation

## Discussion

Implementation of lower, controlled and personalised pressures with automatic pneumatic tourniquet technology has improved the safety, accuracy and reliability of surgical tourniquet systems. Central to this development is the capacity to accurately measure LOP, from which a personalised tourniquet cuff pressure can be recommended. The use of personalised tourniquet settings based on LOP has been limited by the practical difficulties of the manual Doppler ultrasound method, and the limitations of the distal LOP method of automatic LOP measurement. The embedded LOP method of automatic LOP measurement provides several practical advantages, and has been shown to have surgically acceptable accuracy to that of the manual Doppler ultrasound method [12]. The aim of this study was to investigate whether the embedded and distal methods of LOP measurement have clinically acceptable agreement. The results show that LOPs measured with the embedded and distal methods demonstrate clinically acceptable agreement, thereby supporting the broader clinical usage use of the embedded LOP method in a clinical setting.

The accuracy of both the embedded LOP and distal LOP methods has previously been compared to the manual Doppler ultrasound method [12, 18]. McEwen et al. [18] compared the accuracy of the distal LOP method to the manual Doppler ultrasound method. Across 39 measurement pairs in the lower limbs of 20 apparently healthy individuals, the mean difference between the two methods was 1.7 ± 8.9 mmHg, demonstrating that the distal LOP method has clinically acceptable agreement. More recently, Masri et al. [12] compared the accuracy of the embedded LOP method to the manual Doppler ultrasound method. Across 249 measurement pairs in 143 individuals, the mean difference between the two methods was 1 ± 8 mmHg for the upper limbs (*n* = 134), 0 ± 15 mmHg for the lower limbs (*n* = 118), and 1 ± 12 mmHg overall (*n* = 252). The authors concluded that the embedded LOP method has clinically acceptable accuracy with the manual Doppler ultrasound method. The SD of 8.9 mmHg for the mean difference in LOP measurement in the lower limbs in the study by McEwen et al. [18] is slightly lower than the SD of 10.47 mmHg for the lower limbs in the present study. However, the sample size for lower limb measurement pairs in the present study is much greater than the 39 measurement pairs in the study by McEwen et al. [18]. Masri et al. [12] reported that the SDs of the mean differences between the embedded LOP method and manual Doppler ultrasound method were 8 mmHg, 15 mmHg and 12 mmHg for the upper limb, lower limb and combined measurements, respectively. In the present study, the SDs of the mean differences between the embedded LOP and distal LOP methods of LOP measurement were 7.54 mmHg, 10.47 mmHg and 10.35 mmHg for the upper limb, lower limb and combined measurements, respectively. These are comparable or slightly smaller than the SDs of mean differences reported by Masri et al. [12], despite a greater number of measurements in the present study.

It is of note that the bias, 95% of the bias, SD of differences and LOA were greater in the lower limb compared to the upper limb, which may be explained by inherent differences in the LOP measurement methods. The distal method of LOP measurement measures the physiologic signal distally at the digits of the limb and is therefore affected by poor peripheral circulation. Healthy, disease-free individuals generally have better peripheral circulation in the upper limbs. Consequently, there is likely to be less variation in the distal measurement of LOP in the upper compared to the lower limb, which would contribute to smaller differences and narrower CIs and LOAs. Importantly, this observation is similar to the results of Masri et al. who observed narrower CIs in the upper limb compared to the lower limb. Furthermore, this may provide some explanation as to why there was a small negative bias on the upper limb (distal LOP higher than embedded LOP) and a small positive bias on the lower limb (distal LOP lower than embedded LOP). As the two methods of LOP measurement measure LOP differently, it is expected that there will be differences in the results depending on the situation. However, these differences are minor and embedded and distal methods of LOP measurement were found to have clinically acceptable agreement.

The results of the present study demonstrate that the embedded and distal methods of LOP measurement have clinically acceptable agreement. In conjunction with previous studies showing that the embedded LOP and distal LOP methods of automatic LOP measurement have clinically acceptable accuracy with the manual Doppler ultrasound method [12, 18], the present study provides evidence to support the use of embedded LOP method of automatic LOP measurement using dual-purpose tourniquet cuffs in a clinical setting. There are many limitations to the distal LOP and manual Doppler ultrasound methods, which are diminished with the embedded LOP method of automatic LOP measurement. For example, a distal sensor is not required, perioperative workflow and time are less affected as the embedded LOP method allows measurement of LOP while the limb is elevated and being prepared for surgery, the sterile surgical field is unaffected, and the embedded LOP method is not dependent upon peripheral variables that can affect measurement of blood flow distal to the cuff (i.e. poor peripheral circulation) [12, 16].

The present study has important strengths in comparison to previous studies [12, 18]. This includes the repeated pairs of measurements in both the upper and lowers limbs and a larger sample size. Furthermore, our study is the first to directly examine whether the embedded and distal methods of LOP measurement have clinically acceptable agreement. A limitation of our study is the inclusion of only apparently healthy individuals. However, given that the SDs of the mean differences in LOP measurement in the present study are comparable to the SDs reported by Masri et al. in pre- and post-surgical patients [12], we are confident that this does not influence our results.

The findings of this study demonstrate that the embedded and distal methods of LOP measurement have clinically acceptable agreement. The findings thus support the use of the embedded LOP method of automatic LOP measurement using dual-purpose tourniquet cuffs to enable accurate, effective and simple prescription of personalised tourniquet cuff pressures in a surgical and clinical setting.

## Data Availability

The datasets generated and analysed during the current study are not available due to ethical confidentiality but are available from the corresponding author on reasonable request.

## Abbreviations

LOP: Limb Occlusion Pressure
LOA: Limits of Agreement
CI: Confidence Intervals
